# Microbial products linked to steatohepatitis are reduced by deletion of nuclear hormone receptor SHP in mice

**DOI:** 10.1016/j.jlr.2023.100469

**Published:** 2023-11-02

**Authors:** Ryan Mifflin, Jung Eun Park, Mikang Lee, Prasant Kumar Jena, Yu-Jui Yvonne Wan, Hazel A. Barton, Mirjavid Aghayev, Takhar Kasumov, Li Lin, Xinwen Wang, Robert Novak, Feng Li, He Huang, Leah P. Shriver, Yoon-Kwang Lee

**Affiliations:** 1 Department of Integrative Medical Sciences, Northeast Ohio Medical University, Rootstown, OH, USA; 2 Department of Medical Pathology and Laboratory Medicine, University of California, Davis, Sacramento, CA, USA; 3 Department of Biology, University of Akron, Akron, OH, USA; 4 Department of Pharmaceutical Sciences, Northeast Ohio Medical University, Rootstown, OH, USA; 5 Department of Pathology, Northeast Ohio Medical University, Rootstown, OH, USA; 6 Department of Pathology and Immunology, Baylor College of Medicine, Houston, TX, USA; 7 Shanghai Key Laboratory of Metabolic Remodeling and Health, Institute of Metabolism and Integrative Biology, Fudan University, Shanghai, China; 8 Department of Chemistry & Department of Medicine, Center for Metabolomics and Isotope Tracing, Washington University, St. Louis, MO, USA

**Keywords:** bile acids and salts, inflammation, intestine, liver, nuclear receptor/RXR, PPARs, microbiome, lipopolysaccharide, phenylacetic acid, small heterodimer partner

## Abstract

Deletion of the nuclear hormone receptor small heterodimer partner (*Shp*) ameliorates the development of obesity and nonalcoholic steatohepatitis (NASH) in mice. Liver-specific SHP plays a significant role in this amelioration. The gut microbiota has been associated with these metabolic disorders, and the interplay between bile acids (BAs) and gut microbiota contributes to various metabolic disorders. Since hepatic SHP is recognized as a critical regulator in BA synthesis, we assessed the involvement of gut microbiota in the antiobesity and anti-NASH phenotype of *Shp*^*−/−*^ mice. *Shp* deletion significantly altered the levels of a few conjugated BAs. Sequencing the 16S rRNA gene in fecal samples collected from separately housed mice revealed apparent dysbiosis in *Shp*^*−/−*^ mice. Cohousing *Shp*^*−/−*^ mice with WT mice during a Western diet regimen impaired their metabolic improvement and effectively disrupted their distinctive microbiome structure, which became indistinguishable from that of WT mice. While the Western diet challenge significantly increased lipopolysaccharide and phenylacetic acid (PAA) levels in the blood of WT mice, their levels were not increased in *Shp*^*−/−*^ mice. PAA was strongly associated with hepatic peroxisome proliferator-activated receptor gamma isoform 2 (*Pparg2*) activation in mice, which may represent the basis of the molecular mechanism underlying the association of gut bacteria and hepatic steatosis. *Shp* deletion reshapes the gut microbiota possibly by altering BAs. While lipopolysaccharide and PAA are the major driving forces derived from gut microbiota for NASH development, *Shp* deletion decreases these signaling molecules via dysbiosis, thereby partially protecting mice from diet-induced metabolic disorders.

Nuclear hormone receptors play crucial roles in many mammalian physiologies, including metabolic homeostasis, by regulating target gene expression (1). An orphan nuclear hormone receptor, small heterodimer partner (SHP/NR0B2), was identified as an interacting protein of the nuclear hormone receptor constitutive androstane receptor (NRLI3) using a yeast two-hybrid screening strategy (2). Earlier molecular studies found that SHP transcriptionally repressed target gene expression by directly recruiting DNA-bound transcription factors (3, 4, 5). Feedback regulation of cholesterol 7α-hydroxylase (*Cyp7a1*) gene expression by bile acid (BA) has been identified as one of SHP’s most important and widely studied physiological roles and has been a stepping stone for understanding BA and cholesterol metabolism (6, 7). The generation of *Shp*^*−/−*^ mice confirmed the important role of SHP in BA synthesis (8). Subsequent studies using mouse models, including *Shp*^*−/−*^ mice, have further identified the potential role of SHP in lipid and glucose metabolism (9, 10, 11). These studies suggested that *Shp* deletion protects mice from diet-induced obesity and fatty liver but not from diabetes. Suppression of peroxisome proliferator-activated receptor gamma isoform 2 (*Pparg2*) expression was suggested as one proposed molecular mechanisms for attenuating hepatic lipid accumulation in *Shp*^*−/−*^ mice (12). Our accompanying study additionally revealed that *Shp* deletion ameliorates nonalcoholic steatohepatitis (NASH) development induced by a long-term Western diet (WD) (13).

Obesity and nonalcoholic fatty liver disease (NAFLD) are highly associated metabolic disorders impacting modern society. Recent progress in understanding the underlying mechanisms of these metabolic disorders has strongly implicated gut microbiota in their development (14, 15). Studies on ob/ob mice have provided a mechanistic basis for the association between obesity and gut microbiota, where microbiome enrichment with an increased capacity for dietary energy harvesting was evident (16). Transplantation of this obesity-prone microbiota into germ-free mice resulted in a significantly greater body fat increase than transplantation of microbiota from lean control mice, indicating that the microbiota carries determinant factors for metabolic outcomes in its host, which has also been shown in humans (17). In addition, a high-fat diet (HFD) chronically elevates serum bacterial lipopolysaccharide (LPS) by altering gut flora and intestinal permeability, triggering inflammatory signatures and metabolic dysregulations, eventually leading to the phenotypic presentation of obesity, diabetes, and NAFLD (18).

Dietary fat and BAs play critical roles in modulating the gut microbiota. Taurocholic acid induced by saturated fat consumption is directly involved in an expansion of a low-abundance, sulfite-reducing pathobiont, *Bilophila wadswothia*, which is associated with colitis development in interleukin 10^*−/−*^ mice (19). Farnesoid X receptor knockout (*Fxr*^*−/−*^) mice, the primary regulator in BA synthesis, developed many metabolic dysfunctions because of BA-induced alterations in gut microbial composition rather than directly from *Fxr* deletion (20). The alteration of microbial ecology by BA, diet, or xenobiotics can induce intestinal tauro-β-muricholic acid in mice or glycoursodeoxycholic acid in humans, inhibiting intestinal FXR activity and thereby improving various metabolic dysfunctions in the associated diseases (21, 22, 23).

As discussed previously, the orphan nuclear receptor SHP is directly involved in regulating the expression of enzymes involved in the BA synthesis pathway, with its deletion modifying hepatic BA composition (11). To assess the association of gut microbiota with the phenotypes of *Shp*^*−/−*^ mice reported in earlier studies, including an accompanying report (13), we examined the gut microbiota using 16S rRNA gene sequencing of fecal samples from WT and mutant mice and explored phenotypes using mixed-group housing (cohousing) strategies, which enable the sharing of gut microbiota between the two mouse strains (24). Because of BA composition changes, *Shp*^*−/−*^ mice formed bacterial operational taxonomy units (OTUs) distinct from WT mice. Cohousing disrupted the OTU distinctiveness and protective phenotypes against diet-induced obesity and NASH development of *Shp*^*−/−*^ mice in segregated-group (separated) housing. *Shp*^*−/−*^ mice housed in separate cages maintained reduced serum levels of LPS and phenylacetic acid (PAA), a bacterial metabolite highly associated with hepatic fat accumulation in humans, under a WD compared with WT counterparts (25). Moreover, PAA signaling appeared to be associated with hepatic *Pparg2* expression, promoting hepatic lipid storage (26).

## Materials and Methods

### Mouse studies

The *Shp*^*−/−*^ mice have been previously described (27). Only male mice were used in the experiments. The cohousing experiment used equal numbers of age-matched WT C57BL/6NHsd and *Shp*^*−/−*^ mice placed in a cage immediately after weaning. Each genotype was produced by homozygous (nonlittermate) or heterozygous (littermate) breeding. All cages were housed in a temperature- and light-controlled room with a 12/12 h light-dark cycle (on: 06:30, off: 18:30). Food and water were available ad libitum. Rodent chow was obtained from Lab Diet (MO; catalog no.: 5008). In order to induce obesity and NASH, mice were fed a WD (catalog no.: TD.88137; Envigo, IN; 21.2% [w/w] total lipid [42% kcal], 48.5% [w/w] carbohydrates [42.7% kcal], 17.3% [w/w] proteins [15.2% kcal], and 0.2% cholesterol; total = 4.5 kcal/g) starting at 8 weeks old for 24 weeks. Body weight (BW) was measured every week after WD feeding began. WT and *Shp*^*−/−*^ mice in littermate mouse experiments were produced by mating heterozygous *Shp*^*+/−*^ mice. In these experiments, cohousing used WT and *Shp*^*−/−*^ littermates. They were fed the same diet regimen described above. Tissues were collected after overnight fasting at the end of the diet regimen. Fecal samples were collected twice, immediately before and after 10 weeks of the diet regimen, for 16S rRNA gene sequencing. After 14 weeks of the WD, mouse body composition was determined using an EchoMRI machine (EchoMRI, TX). All animal care and use protocols were approved by the Institutional Animal Care and Use Committee of Northeast Ohio Medical University (Rootstown, OH).

### Lipidomics

Lipids were extracted from approximately 20 mg of liver in a chloroform-methanol mixture. The extracted lipids were analyzed using shotgun lipidomics as previously described (28). Protein layers from chloroform-methanol extraction were used to normalize lipid quantities as ion counts based on the obtained protein concentrations.

### BA analysis

BAs were extracted from 5 μl of bile from the gallbladder (GB) and of weighed ileum with luminal content as previously described (29). BAs were analyzed in the samples from separately housed mice using LC-MS/MS (6490 Triple Quadrupole LC/MS; Agilent Technologies, Santa Clara, CA) at Baylor College of Medicine (Houston, TX). For GB samples from cohoused mice, 1 μl of bile was diluted with 99 μl of methanol and 10 μl of internal standards containing 2 μg/ml of LCA-d4 and CA-d4 in methanol. The BA composition was analyzed using a Q-Exactive Orbitrap Mass Spectrometer coupled with an ultraperformance liquid chromatography system (Thermo Fisher Scientific, San Jose, CA) at the Northeast Ohio Medical University.

### RNA isolation and quantitative real-time PCR

The quantitative PCR analysis was performed as previously described in detail (27). Briefly, complementary DNA was synthesized using PrimeScript RT Master Mix (Clontech, CA) from total RNA extracted from the tissues, and quantitative PCR was performed using a CFX96 Real-Time PCR Machine with iTaq Universal SYBR Supermix (BioRad, CA). *Gapdh* was used as the internal control, and relative expression was determined from ΔCt values normalized to the expression of separate WT mice fed a chow diet (CD).

### Gut microbiome profiling and bacterial pathway analysis

Fecal samples were collected from all cages immediately before initiating the WD and again after 10 weeks of the diet regimen (CD or WD). Mice were placed individually into an autoclave-sterilized cage for 36 h with sterilized water, a matching diet, and a bedding. All feces were collected in a laminar flow hood and stored at −80°C until used. Bacterial DNA was extracted using a Fecal DNA MiniPrep Kit (Zymo Research, CA). PCR was performed using primers targeting the V3 and V4 regions of the 16S rRNA gene (Integrated DNA Technologies, IA). The primers are listed in supplemental Table S1. Pippin Prep cassettes (1.5% agarose) were used to purify and collect the targeted DNA (Sage Science, MA). Purified samples were quantified using Qubit spectroscopy (Qubit Systems, ON, Canada) using a double-stranded DNA High-Sensitivity Assay Kit (Thermo Fisher Scientific, NY). The double-stranded DNA amplicons were sent to the Advanced Genetic Technologies Center (University of Kentucky, Lexington, KY) for Illumina MiSeq sequencing on a dual-indexed, 250-base pair flowcell (Illumina, CA). The output files were demultiplexed using QIIME (version 1.9.1, http://www.qiime.org/ inside of the parenthesis), and OTUs were determined using an open-reference algorithm (30, 31). For samples with a sequence count >104,006, beta diversity principal coordinates were estimated using unweighted UniFrac and Adonis Permanova to measure significance (*P* value) and effect size (*R*^2^) to explain variation, and rarefied alpha diversity was estimated using Faith’s phylogenetic diversity whole tree analysis (32, 33). Unless otherwise specified, a homoscedastic Student’s *t-*test was used to compare two groups, with a *P* < 0.05 considered statistically significant. Unless otherwise specified, values are presented as mean ± standard deviation. In order to identify differentially enriched functional pathways from the 16S rRNA gene sequencing data, linear discrimination analysis effect size (LEfSe) was calculated for the Kyoto Encyclopedia of Genes and Genomes orthology functions using the Phylogenetic Investigation of Communities by Reconstruction of Unobserved State (PICRUSt) software package (34, 35). The microbiome analysis of fecal samples from mice obtained via heterozygous mating strategy was performed in EzBiome, Inc (Gaithersburg, MD) as previously described (36).

### Serum PAA measurement

Serum PAA levels were measured in blood collected under isoflurane anesthesia from the portal vein of 4-month-old mice on the diet regimen for 2 months after fasting for 5 h. The serum was processed as previously described (37). PAA was quantified using GC-MS (Agilent Technologies) with phenyl-D5-acetic acid (Sigma, St. Louis, MO) as the internal standard.

### Serum alanine aminotransferase measurement

Serum alanine aminotransferase concentrations in mice on the diet regimen for 6 months were measured colorimetrically (Infinity Reagent; Thermo Fisher Scientific, Waltham, MA) using Synergy 4 (BioTek, Winooski, VT).

### Serum LPS and fecal albumin measurements

Serum LPS concentrations were quantified using an ELISA kit (LSBio, Seattle, WA). Fecal albumin concentrations were quantified in the samples collected from mice at the end of the 6-month WD regimen using an ELISA kit (Immunology Consultation Lab, Inc, Portland, OR). Briefly, about 100 mg of feces were homogenized in 500 μl of phosphate-buffered saline. After centrifugation, supernatants were diluted 400 times before ELISA analysis.

### PAA treatment of a mouse hepatoma cell line

Hepa1-6 cells were seeded into 24-well plates and maintained in Dulbecco’s modified Eagle’s medium until 100% confluent. Next, cells were treated with 10 mM PAA as previously described (25) for 0, 1, 3, and 5 h before being harvested for RNA isolation.

### Statistical analysis

Unless otherwise specified, values are presented as mean ± standard error of the mean. Student’s *t-*test was used to compare two groups. A *P* < 0.05 was considered statistically significant. We also analyzed various datasets using two-way ANOVA. Statistical analyses were performed using GraphPad Prism software (GraphPad Software, Inc) and presented in the associated figures.

## Results

### Metabolic phenotypes of *Shp*^*−/−*^ mice in the cohousing strategy

**Fig. 1:**
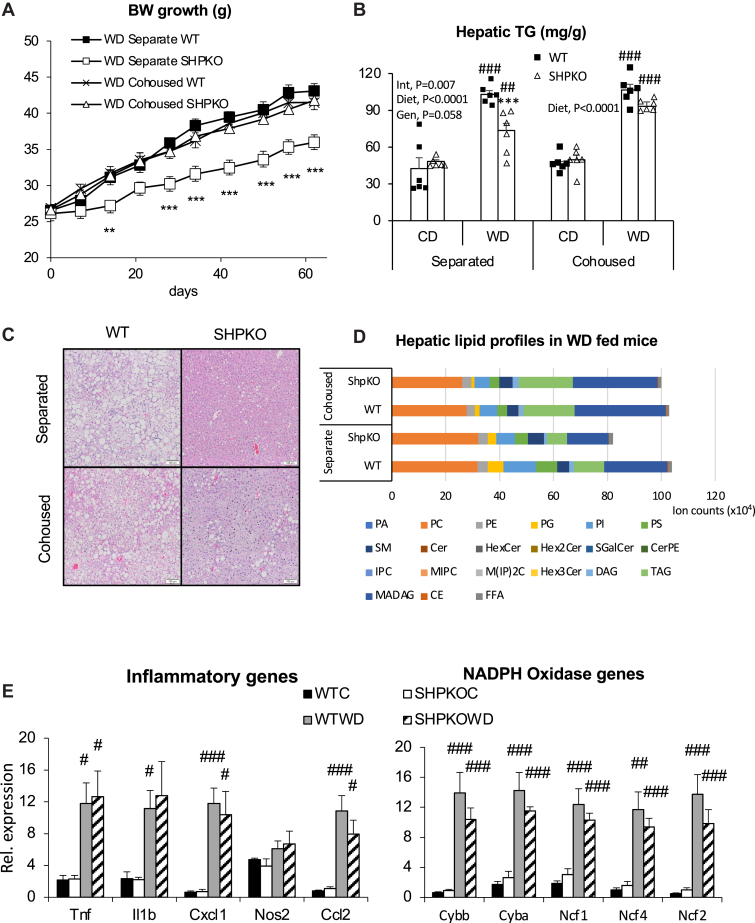
The metabolic phenotypes presented by *Shp* gene deletion are disrupted by cohousing scheme. A: BWs of WT and *Shp*^*−/−*^ mice fed WD from separate or cohousing scheme were measured and plotted with standard errors for the first 2-month regimen. (Separate WT, cohoused WT, cohoused *Shp*^*−/−*^; *n* = 8. Separate *Shp*^*−/−*^; *n* = 7). B: Hepatic TG concentrations (mg/g of liver weight) were quantified from the WT and *Shp*^*−/−*^ mice fed CD or WD in separate or cohoused cages for 6 months, and their averages were plotted with standard errors (*n* = 6). Two-way ANOVA and Student' *t*-test were performed to evaluate statistical significance. C: Livers from the experimental mice were processed for H&E staining. Representative H&E sections from mice fed WD were shown. White bars at right lower corner represent 100 μm length. D: Extracted lipids from livers of the WD-fed mice were analyzed using LC-MS. Average values of individual lipid class from each group (*n* = 4) were plotted in stacked columns. E: mRNA expression of hepatic genes responsible for inflammation were assessed using quantitative PCR analysis in cohoused WT and *Shp*^*−/−*^ mice and presented with average ± SEM (*n* = 4–6). #comparison with CD, ∗comparison with WT counterparts. Three symbols represent *P* < 0.005, two symbols *P* < 0.01, and one symbol *P* < 0.05. CE, cholesteryl ester; Cer, ceramide; CerPE, ceramide phosphatidylethanolamine; DAG, diacylglycerol; FFA, free fatty acid; HexCer, monohexylceramide; Hex2Cer, dihexylceramide; Hex3Cer, trihexosylceramide; IPC, inositolphosphorylceramide; MADAG, monoalkyldiacylglycerol; MIPC, mannosyl-inositol phosphorylceramide; M(IP)2C, mannosyl-di-(inositolphosphoryl) ceramide; PA, phosphatidic acid; PC, phosphatidylcholine; PE, phosphatidylethanolamine; PG, phosphatidylglycerol; PI, phosphatidylinositol; PS, phosphatidylserine; SGalCer, 3-O-sulfogalatisylceramide; SM, sphingomyelin; TAG, triacylglycerol.

Our earlier studies, including the accompanying report, showed that *Shp* deletion attenuated obesity and NAFLD or NASH development in mice induced by a WD or methionine/choline-deficient diet. A strong association between BA, gut microbiota, and metabolic phenotypes prompted us to assess the involvement of gut microbiota in the phenotypic manifestations observed in *Shp*^*−/−*^ mice. Cohousing strategies have often been used in mouse studies to determine the effect of microbiota on presented metabolic features because of their coprophagic nature (38, 39). Therefore, WT and *Shp*^*−/−*^ mice were cohoused at weaning and subjected to a WD feeding regimen starting from 10 weeks old for 6 months for metabolic assessment. Their BW gains were compared with those of WT and *Shp*^*−/−*^ mice raised in separate cages (Fig. 1A). While BW gain because of WD feeding was attenuated in *Shp*^*−/−*^ mice housed in separate cages, the attenuation was abolished in *Shp*^*−/−*^ mice cohoused with WT mice. In addition, *Shp*^*−/−*^ mice fed a WD and cohoused with WT mice no longer showed significantly lower liver-to-BW ratios (supplemental Fig. S1A). Similarly, triglyceride (TG) concentrations and hematoxylin and eosin staining showed that reduced hepatic TG accumulation was also disrupted in *Shp*^*−/−*^ mice cohoused with WT mice (Fig. 1B, C). Detailed lipid profiles were determined by LC-MS (Fig. 1D), showing a similar pattern to the measured TG levels. Cohousing significantly affected the levels of diacylglycerol, triacylglycerol, monoalkyl diacylglycerol, and cholesteryl ester, which were all significantly lower in separately housed *Shp*^*−/−*^ mice (supplemental Fig. S1B). Mysteriously, however, TG values measured using a commercial kit (Fig. 1B) looked different from those measured using a GS-MS (supplemental Fig. S1B). Because the commercial kit quantifies all glycerols released from the extracted lipid after lipase treatment, the obtained values reflected more closely total lipid values measured by the GC-MS (Fig. 1D).

In our accompanying report, we also assessed inflammatory gene expression to evaluate the potential impact of gut microbiota on the attenuation of the NASH phenotype in *Shp*^*−/−*^ mice (13). Unlike *Shp*^*−/−*^ mice housed in separate cages, *Shp*^*−/−*^ mice fed a WD showed comparable hepatic expression of inflammatory and nicotinamide adenine dinucleotide phosphate oxidase genes to WT mice when cohoused (Fig. 1E), indicating that cohousing almost entirely abolished the phenotypic attenuation by *Shp* deletion.

### BA profiles of *Shp*^*−/−*^ mice

**Fig. 2:**
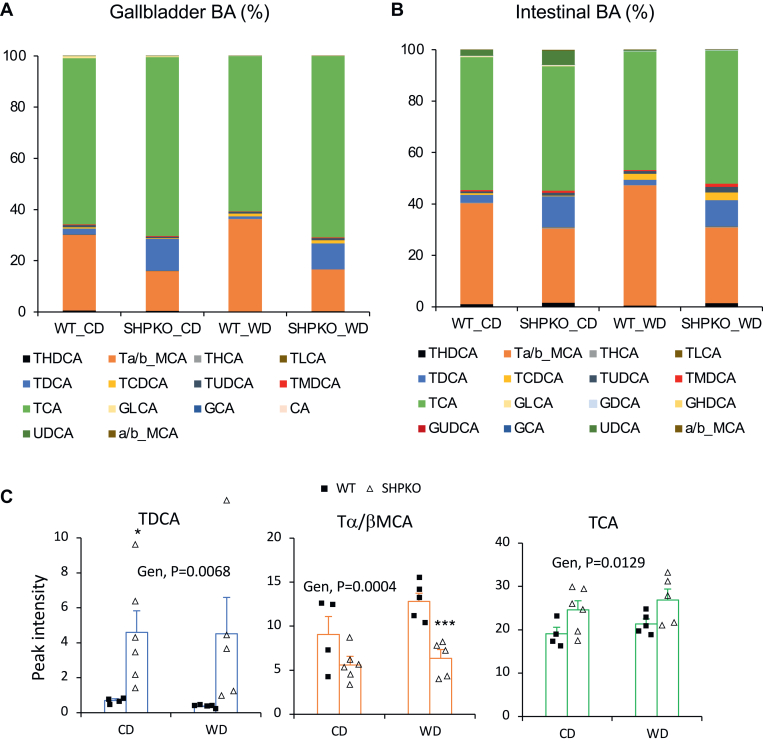
BA compositions in GBs and small intestines from WT and *Shp*^*−/−*^ mice. A: BAs extracted from GBs of experimental animals fed CD or WD were analyzed using an LC–MS. Percent values calculated from their average peak intensities (N; WTC = 4, SHPKOC = 6, WTWD = 5, and SHPKOWD = 5) were plotted into a stacked column graph. B: BAs were extracted from weighed ilea, and their compositions were analyzed. Percent values were calculated from their average peak intensities normalized by weight and were plotted in a stacked column graph (*N* = 6). C: TDCA, TMC, and TCA values from GBs were plotted with average ± SEM. Two-way ANOVA was performed to obtain statistical values. CA, cholic acid; Gen, genotypes; GCA, glycocholic acid; GLCA, glycolithocholic acid; MCA, muricholic acid; TCA, taurocholic acid; TCDCA, taurochenodeoxycholic acid; TDCA, taurodeoxycholic acid; THCA, taurohyocholic acid; THDCA, taurohyodeoxycholic acid; TLCA, taurolithocholic acid; TMCA, tauromurcholic aicd; TMDCA, tauromurideoxycholic acid; TUDCA, tauroursodeoxycholic acid; UDCA, ursodeoxycholic acid.

Based on the observations that SHP is important in BA synthesis, we hypothesized that an altered hepatic BA profile may affect the gut microbiome, thereby modulating metabolic and inflammatory responses in *Shp*^*−/−*^ mice (8, 40). One of our earlier studies showed that the livers of *Shp*^*−/−*^ mice contained higher cholic acid and lower muricholic acid than those of WT mice (11). We extend the earlier study here by reporting their detailed BA profiles, including conjugated BAs (supplemental Fig. S2A). The most abundant hepatic BA in *Shp*^*−/−*^ mice was taurocholic acid (TCA; 230.85 nmol/g), higher than in WT mice (147.86 nmol/g). While total taurine-conjugated BAs were more abundant in *Shp*^*−/−*^ than in WT mice, likely because of their higher expression of cysteine sulfinic acid decarboxylase, a key enzyme in taurine synthesis (41), total muricholic acids and their taurine-conjugated forms were less abundant in *Shp*^*−/−*^ mice probably because of higher *Cyp8b1* expression (supplemental Fig. S7). In this study, we analyzed BA profiles in the GB and intestine, which directly affect the structure of gut microbiota, of the mice fed a CD or a WD (Fig. 2). BA concentrations in the GB and liver of *Shp*^*−/−*^ mice were similarly higher than those in WT mice (19% for CD and 10% for WD). Because of the strong affinity of two major hepatic canalicular membrane BA transporters (bile salt export pump and multidrug resistance-associated protein 2) for conjugated BAs (42), unconjugated BAs were barely detected in the GB from WT and *Shp*^*−/−*^ mice, and the overall conjugated BA profiles were very similar between the liver and GB. However, considering the reported 2.5-fold higher GB volume in *Shp*^*−/−*^ mice than in WT mice (11), the absolute amount of each BA in the GB of *Shp*^*−/−*^ mice would be >2.5 higher than shown in Fig. 2A compared with BAs in WT mice. Deoxycholic acid (DCA), a secondary BA, and taurodeoxycholic acid, its conjugated form, were most significantly increased by *Shp* deletion in the liver (3.5-fold) and GB (6.7-fold or 16.75-fold when the increased bile volume is considered), and β-muricholic acid was the most significantly decreased by *Shp* deletion in the liver and GB (supplemental Fig. S2B, C). Intestinal BA profiles were relatively similar to the liver and GB profiles but with decreased TCA (Fig. 2B), which is supported by an earlier report that TCA is most actively absorbed in the intestine (43). Therefore, the less absorbed tauromuricholic acid appeared to be increased in the intestinal BA profile. In addition, a significant amount of ursodeoxycholic acid, a secondary BA, was detected in the intestine of both WT and *Shp*^*−/−*^ mice (from 0.008% ± 0.002% in the GB of both mouse strains to 2.3% ± 1.3% and 5.6% ± 2.9% in the intestine of WT and *Shp*^*−/−*^ mice, respectively) fed a CD. However, it was significantly reduced with the WD in both mouse strains. The higher overall DCA concentration indirectly indicates structural changes in the gut microbiota of *Shp*^*−/−*^ mice.

### Alteration of gut microbiota in *Shp*^*−/−*^ mice

**Fig. 3:**
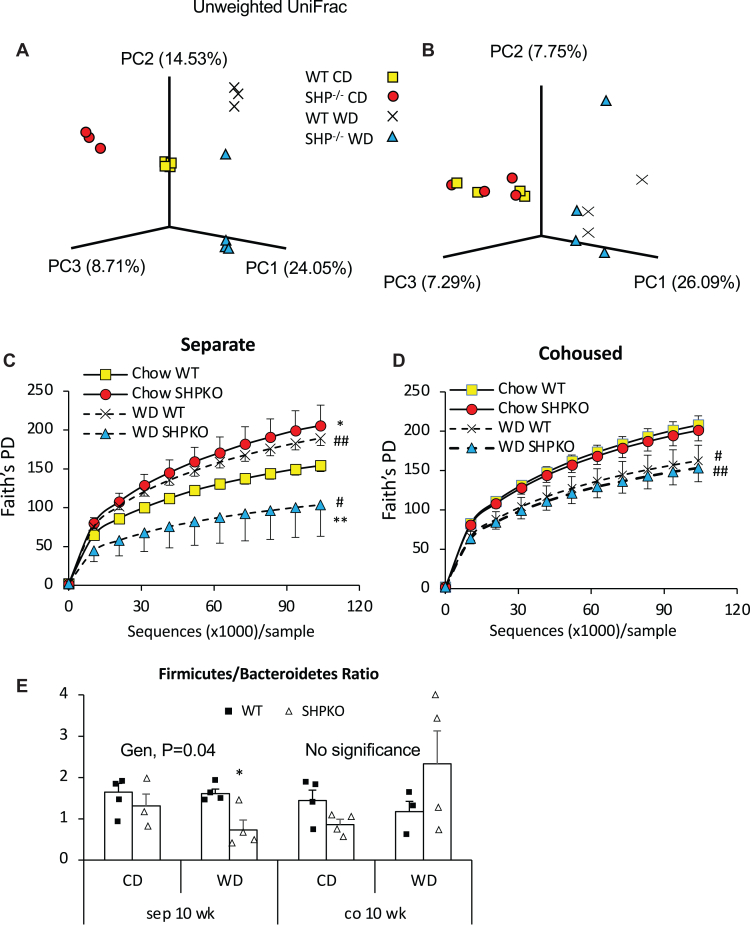
Gut microbiome structure in the experimental mice fed CD or WD. A and B: Three-dimensional principal coordinate analysis was performed with gut bacterial OUTs obtained from fecal samples of animals in separated housing (A) or cohousing (B) using unweighted UniFrac beta-diversity. C and D: Alpha-diversity was assessed with the obtained OTUs using Faith’s phylogenetic diversity analysis (Faith’s PD) in animals housed in separate cages (C) or cohoused cages (D). E: Ratios of *Firmicute*s to Bacteriodetes based on 16S rRNA gene sequencing data were plotted with average ± SEM (*n* = 3–4).

To evaluate the potential impact of *Shp* deletion on gut microbiota composition, we sequenced the 16S rRNA gene in fecal DNA from WT and *Shp*^*−/−*^ mice fed a CD or a WD for 10 weeks. First, we performed principal coordinate analysis using unweighted UniFrac distances between the OTUs obtained from the 16S rRNA gene sequencing to assess β-diversity in each group. Figure 3A shows that each group forms a distinctive cluster depending on their genotype and diet, suggesting that the gut bacterial composition of *Shp*^*−/−*^ mice differs significantly from that of WT mice. Faith’s phylogenetic diversity did not differ significantly between mouse strains at a young age before the diet intervention (supplemental Fig. S3A). At 16 weeks of age, phylogenic diversity increased significantly in *Shp*^*−/−*^ mice fed a CD. However, 10 weeks of a WD significantly decreased phylogenic diversity in *Shp*^*−/−*^ mice compared with WT mice (Fig. 3C). At the phylum level, the WD increased *Proteobacteria* in both mouse strains. Diet-induced obesity has been reported to increase the abundance of Firmicutes and decrease the abundance of Bacteroidetes (44). The WD decreased Firmicutes and increased Bacteroidetes in *Shp*^*−/−*^ mice, significantly decreasing their ratio (Fig. 3E and supplemental Fig. S3B).

Cohousing disrupted the clear separation of gut bacterial OTUs observed in the separately caged groups and the difference in phylogenic diversity with maintained dietary effect (Fig. 3B, D). The ratio of Firmicutes to Bacteroidetes was significantly increased in cohoused *Shp*^*−/−*^ mice fed the WD, which showed an obese phenotype (Fig. 3E). We also assessed the bacterial populations in each group (supplemental Fig. S4). Their abundance did not differ between the two mouse strains, although a stronger bactericidal effect was expected in *Shp*^*−/−*^ mice with higher BA pool size (40).

**Fig. 4:**
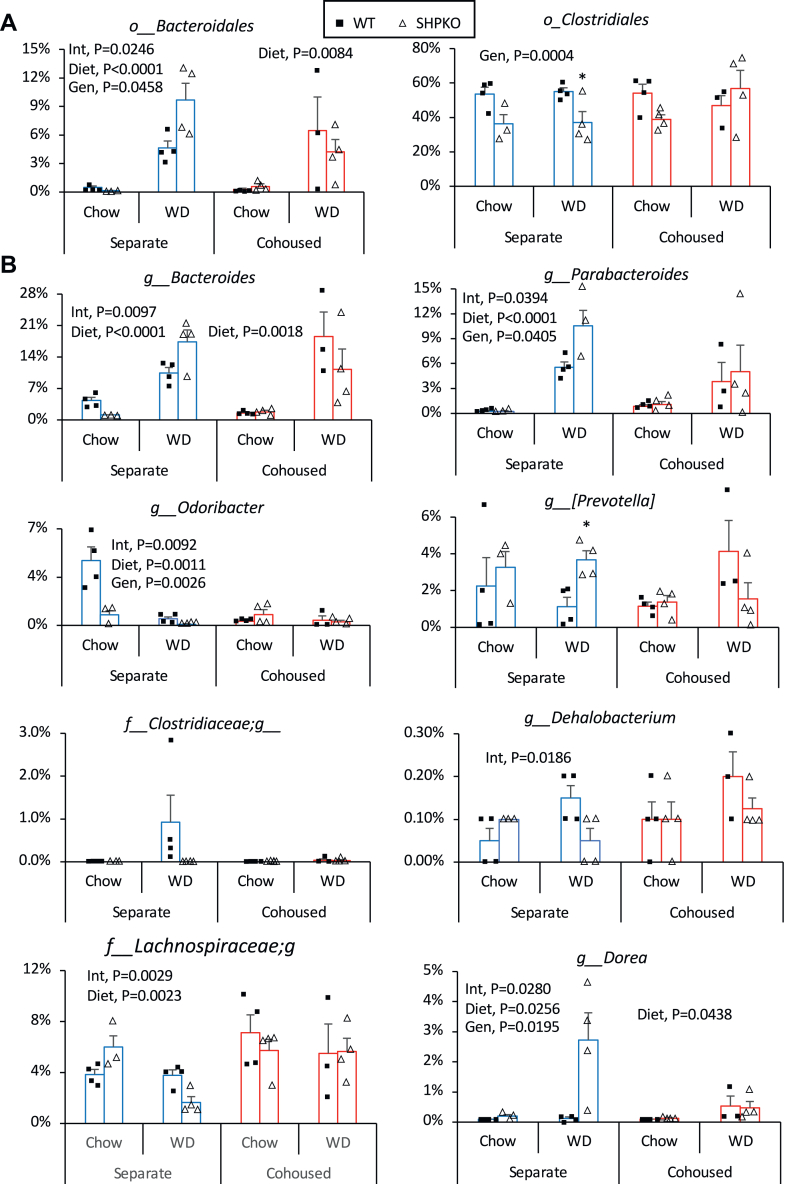
Gut microbes in *Shp*^*−/−*^ mice affected by cohousing. Phylogenetic gut microbes were classified based on fecal 16S rRNA gene sequencing. Their abundances presented as percent values were compared between groups studied in Fig. 3A. Orders of Bacteriodales (left) and Clostridales (right) were presented as percent abundance ± SEM between experimental groups. B: Eight genera including unclassified ones belonging to the two orders were presented as bar graphs based on their average percent abundances with SEM. Two-way ANOVA results were shown with *P* values. Gen, genotype; Int, interaction.

Our 16S rRNA gene sequencing identified gut bacteria up to the genus level. Of the screened 174 genus-level OTUs, 53 had at least two decimal percent values in any mouse. Comparison of these 53 genera between the separate and cohoused groups showed that the two most abundant bacterial orders, Bacteroidales and Clostridiales, were significantly impacted by cohousing in *Shp*^*−/−*^ mice (Fig. 4A). Among the genera in the order Bacteroidales, the abundances of Bacteroides, Parabacteroides, Odoribacter, and Prevotella were significantly altered in *Shp*^*−/−*^ mice fed a WD compared with WT mice (Fig. 4B). In the order Clostridiales, the abundances of unidentified genera in the family Clostridiaceae and Lachnospiraceae and genera Dehalobacterium and Dorea differed significantly between WT and *Shp*^*−/−*^ mice fed a WD. However, these differences were disrupted by cohousing.

We also assessed the functional pathways of the microbial communities using PICRUSt and identified differentially enriched pathways using LEfSe analysis (34). The top enriched pathways and their numbers (LEfSe ≥2) in each group were considered in three categories (genotype, diet, and housing; Table 1). The highest number of enriched pathways (61) were found in the separately housed CD-fed genotype category. The number of enriched pathways decreased to 17 when the cohoused group of the same category was analyzed. The smallest number of pathways (3) were enriched when comparing the separate and cohoused WT WD-fed groups. In contrast, the same comparison of *Shp*^*−/−*^ WD groups identified 45 enriched pathways. This observation suggests that cohousing greatly affected the gut microbiome of *Shp*^*−/−*^ mice compared with WT mice. While only 12 pathways were enriched between cohoused WT and *Shp*^*−/−*^ mice fed the WD, these results indicate that cohousing modifies the gut microbiota of *Shp*^*−/−*^ mice to become similar to WT mice, consistent with the described phenotypic outcomes. Some of the pathways positively associated with human obesity were enriched in the separately housed WT WD group, and some negatively associated pathways were enriched in the separately housed *Shp*^*−/−*^ WD group, confirming the validity of this analysis (supplemental Fig. S5) (16).

**Table 1:** Differentially enriched microbial pathways in each group predicted from fecal 16S RNA gene sequencing

Group	Number of pathways & top four enriched pathways in each group				Total
Genotype		WT		ShpKO	
Separate CD	18	Transporters	43	Ribosome	61
		Bacterial motility proteins		DNA repair and recombination proteins	
		Flagella assembly		Phosphotransferase system	
		Two-component system		Mismatch repair	
Separate WD	12	Transporters	22	Citrate cycle—tricarboxylic acid cycle	34
		Bacterial motility proteins		Carbon fixation pathways in prokaryotes	
		Betalain biosynthesis		Oxidative phosphorylation	
		Flagella assembly		Ala, Asp, and glutamate metabolism	
Cohoused CD	6	Bacterial motility proteins	11	Carbon fixation in photosynthetic organisms	17
		Flagella assembly		Nucleotide metabolism	
		Bacterial chemotaxis		Bacterial secretion system	
		Starch and sucrose metabolism		Cell mobility and secretion	
Cohoused WD	12	Pyrimidine metabolism	0		12
		Peptidases			
		Purine metabolism			
		Ribosome			

Diet		CD		WD	
Separate WT	5	Peptidases	16	Other ion-coupled transporters	21
		Streptomycin biosynthesis		Cysteine and methionine metabolism	
		Other glycan degradation		Replication recombination repair proteins	
		Drug metabolism other enzymes		Restriction enzyme	
Separate ShpKO	3	Phosphotransferase system	12	Other ion-coupled transporters	15
		Aminoacyl tRNA biosynthesis		Pyruvate metabolism	
		*Staphylococcus aureus* infection		Carbon fixation in photosynthetic organisms	
				Inorganic ion transport and metabolism	
Cohoused WT	4	Methane metabolism	5	General function prediction only	9
		Lysine biosynthesis		Other ion-coupled transporters	
		C5 branched dibasic acid metabolism		Amino sugar and nucleotide sugar metabolism	
		DDT degradation		Inorganic ion transport and metabolism	
Cohoused ShpKO	35	Ribosome	11	Two-component system	46
		DNA repair and recombination proteins		Others	
		Amino acid–related enzymes		Function unknown	
		Pyrimidine metabolism		Secretion system	

Housing		Separate		Cohoused	
WT CD	7	Protein folding and associated process	10	Phe, Tyr, and tryptophan biosynthesis	17
		Function unknown		Cysteine and methionine metabolism	
		Other glycan degradation		Methane metabolism	
		Amino sugar and nucleotide sugar metabolism		Nitrogen metabolism	
ShpKO CD	0		5	Val, Leu, and isoleucine biosynthesis	5
				Cell motility and secretion	
				Phenylalanine metabolism	
				Geraniol degradation	
WT WD	1	Methane metabolism	2	Photosynthesis proteins	3
				Starch and sucrose metabolism	
ShpKO WD	34	Ribosome	11	Two-component system	45
		Energy metabolism		Function unknown	
		Ala, Asp, and glutamate metabolism		Secretion system	
		DNA replication proteins		Others	

PICRUSt and LEfSe analyses were performed to predict enriched microbial pathways in each experimental group from fecal 16S RNA sequencing results described in Fig. 3.

### Serum LPS and phenylacetate responsible for NASH development are reduced in *Shp*^*−/−*^ mice fed the WD

**Fig. 5:**
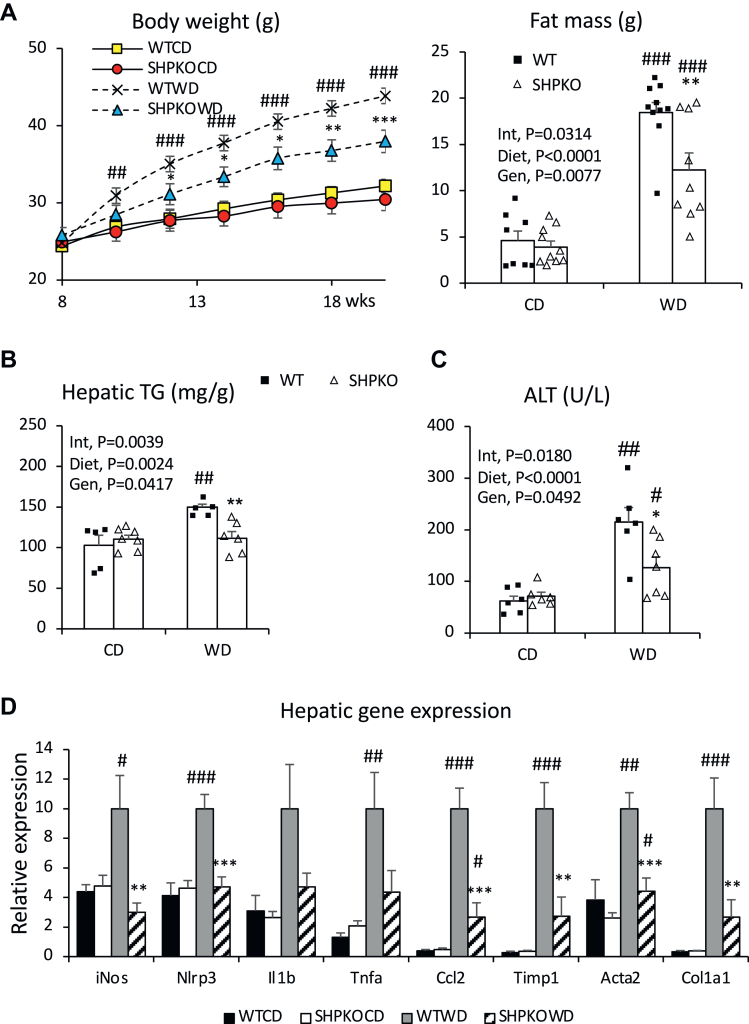
Metabolic features in *Shp*^*−/−*^ mice generated from heterozygous breeding scheme. A: Their body weights upon WD regimen were monitored, and the average values were plotted with SEM (left). Their fat masses were assessed using EchoMRI one day before euthanasia for tissue collection (right). The average values were presented with SEM. *n* = 8–10. B: Hepatic TG levels were presented as means ± SEM. *n* = 5–6. C: Serum ALT levels were assessed, and their average values were presented with SEM. *n* = 6–7. D: mRNA levels of indicated hepatic inflammatory and fibrogenic genes were quantified, and their mean values were plotted with SEM (*n* = 6–8). Two-way ANOVA and Student's *t*-test were performed to evaluate statistical significance. #comparison with CD, ∗comparison with WT counterparts. Three symbols represent *P* < 0.005, two symbols *P* < 0.01, and one symbol *P* < 0.05.

Gut dysbiosis and disease phenotypes manifested in earlier *Nlrp* inflammasome gene knockout mouse studies (45, 46), which used nonlittermate control WT mice, were recently challenged by studies using littermate mice (47, 48). To determine whether cage or maternal effects contributed to the observed phenotypes in *Shp*^*−/−*^ mice, biochemical and physiological features were reassessed in *Shp*^*−/−*^ mice generated from heterozygous breeding and compared with their WT littermates. Figure 5 shows that all the metabolic phenotypes and gene expression patterns in littermate WT and *Shp*^*−/−*^ mice were almost identical to those in nonlittermate studies. These observations suggest that phenotypes manifested by separately housed and cohoused *Shp*^*−/−*^ mice were not caused by housing or maternal inheritance but by *Shp* gene deletion, which changes the BA profile and microbial ecosystem in the gastrointestinal tract. In addition, structures of gut microbes from these two genotypes generated by heterozygous breeding were clearly separated in principal coordinate analysis based on 16S rRNA gene sequencing data regardless of their diet (supplemental Fig. S6).

**Fig. 6:**
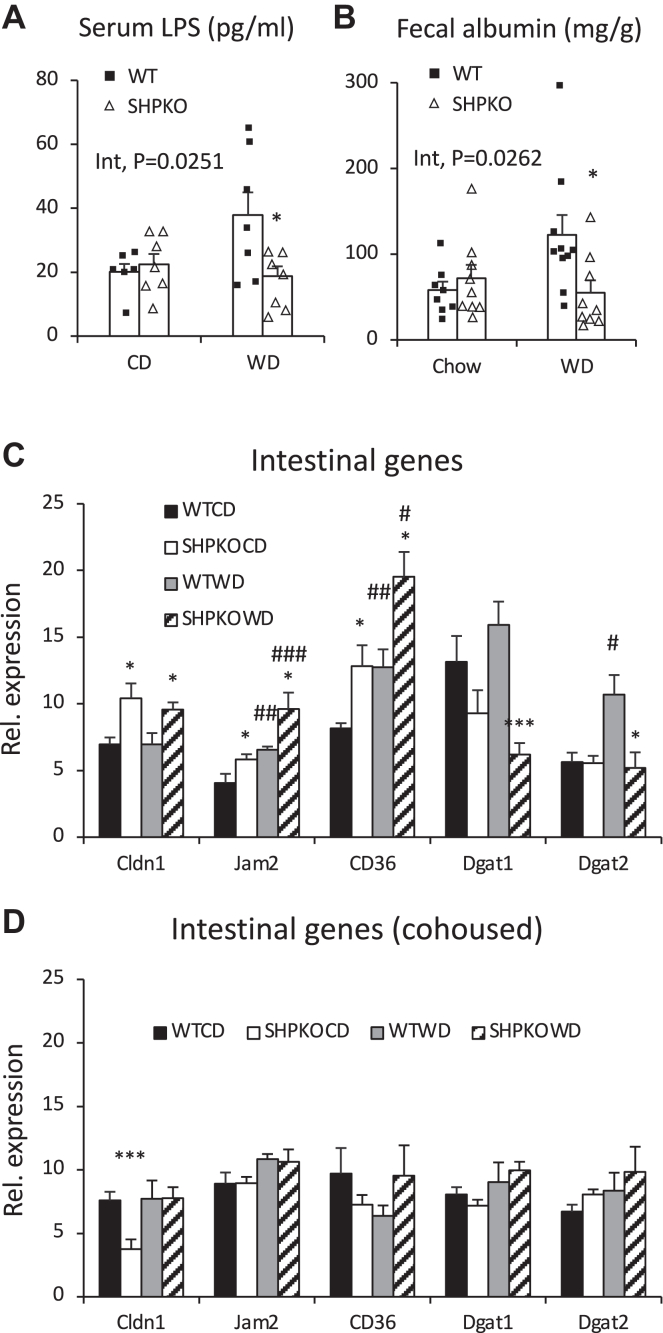
Reduced endotoxemia and gut permeability in *Shp*^*−/−*^ mice fed WD. A: LPS levels were measured in serum collected from WT and *Shp*^*−/−*^ mice generated by heterozygous mating. Their average values were presented with SEM (*n* = 6–7). B: Fecal albumin levels in the experimental animals were quantified and plotted as means ± SEM (*n* = 8–10). C and D: quantitative PCR analysis was performed to quantify mRNA levels of indicated genes involved gut permeability and fat absorption in their small intestines from separately housed (C) and cohoused mice (D). *n* = 5–8. Two-way ANOVA and Student's *t*-test were performed to evaluate statistical significance. #comparison with CD, ∗comparison with WT counterparts. Three symbols represent *P* < 0.005, two symbols *P* < 0.01, and one symbol *P* < 0.05.

Endotoxemia has been considered one of the major contributors causing diet-associated metabolic diseases such as obesity and fatty liver (18). In order to test the involvement of LPS in phenotypic manifestations, serum LPS levels were measured in the littermates of WT and *Shp*^*−/−*^ mice fed a CD or a WD. Figure 6A shows that LPS levels increased approximately 2-fold after the WD challenge in WT mice but not in *Shp*^*−/−*^ mice, causing LPS levels to differ significantly between WT and *Shp*^*−/−*^ on the WD regimen. Since higher LPS penetration into the circulation can result from increased intestinal permeability, mRNA levels of genes involved in intestinal permeability were examined. The mRNA levels of claudin 1 (*Cldn1*), junction adhesion molecule 2 (*Jam2*), and cluster of differentiation 36 (*Cd36*) were significantly higher in *Shp*^*−/−*^ mice than in WT mice fed a WD (Fig. 6C). Since the deletion of *Cd36* in endothelial cells affected gut permeability via extracellular matrix remodeling (49), we assessed fecal albumin levels, an indicator of intestinal barrier disruption, in our mice (50). Like serum LPS levels, fecal albumin concentrations were significantly lower in WD-fed *Shp*^*−/−*^ mice than in WT mice but were similar in CD-fed WT and *Shp*^*−/−*^ mice (around 60 ng/mg; Fig. 6B). While increased endotoxemia was attributed to increased serum alanine aminotransferase levels in WT mice fed a WD, these diet-induced insults were attenuated in *Shp*^*−/−*^ mice (Fig. 5C).

In addition to the genes directly involved in gut barrier function, a few genes involved in fatty acid esterification were also examined. Among those genes, diacylglycerol O-acyltransferase 1 (*Dgat1*) and 2 (*Dgat2*) expression was significantly downregulated in WD-fed *Shp*^*−/−*^ mice compared with WT mice (Fig. 6C). The expression of these two genes was reported to be associated with specific intestinal microbes in response to an HFD for fat absorption (51). Since many genus-level bacteria belonging to the family Clostridiaceae were reported to be responsible for the overexpression of these genes and dietary lipid absorption, this observation indicated an altered gut microbiome (especially the data in Fig. 4B) and impaired intestinal fat absorption in *Shp*^*−/−*^ mice (52).

**Fig. 7:**
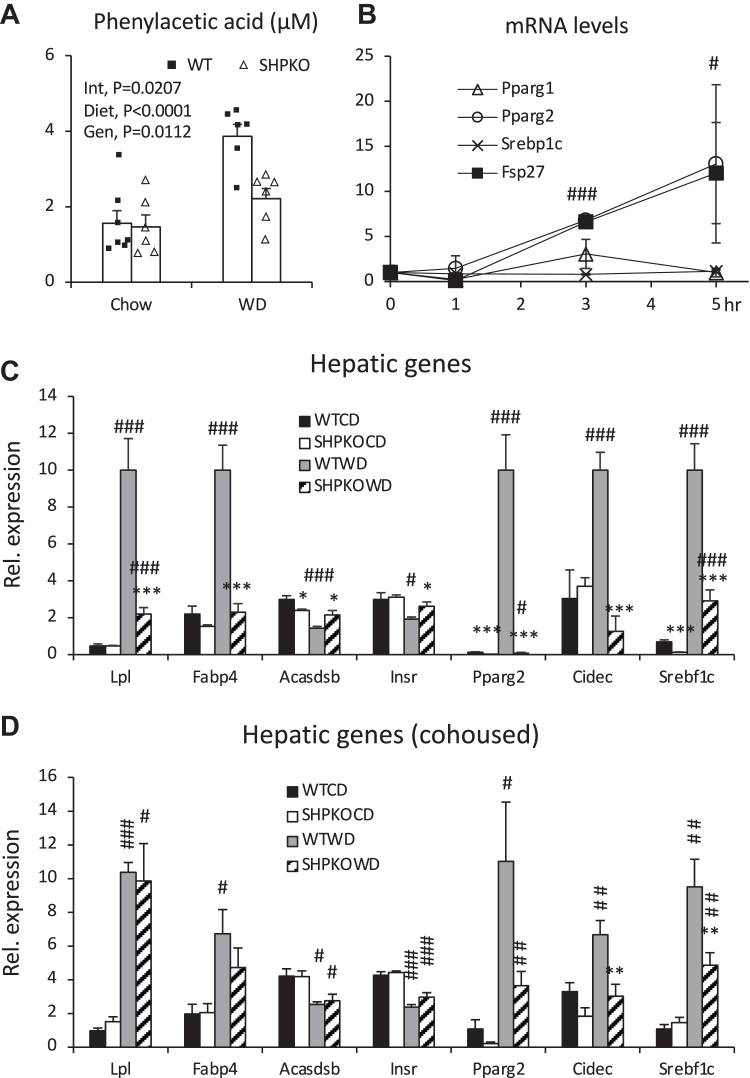
Levels of PAA in portal veins and its association with hepatic PPARG2 activation. A: PAA levels were determined in the blood collected from portal veins using a GC-MS. Their averages were presented with SEM (*n* = 6–7). B: Hepa1-6, a mouse hepatoma cell line, cells were treated with PAA for indicated time points. Levels of mRNA of the indicated genes were quantified by quantitative PCR analysis from triplicates. Statistical results are from comparison of both *Pparg2* and *Fsp27* with 0 time points (*n* = 3). C and D: Expression of hepatic genes associated with microbial PAA was assessed in WT and *Shp*^*−/−*^ mice fed CD or WD from separately grouped (C) and cohoused strategies (D) using quantitative PCR analysis (*n* = 5–8). Two-way ANOVA or Student's *t*-test was performed to evaluate statistical significance. #comparison with CD, ∗comparison with WT counterparts. Three symbols represent *P* < 0.005, two symbols *P* < 0.01, and one symbol *P* < 0.05.

A recent integrative multiomics study on the molecular mechanisms responsible for the integrated interactions between signals from gut bacteria and host hepatic steatosis identified serum PAA as the strongest positive marker of hepatic steatosis among microbial-mammalian cometabolites in women with morbid obesity (25). Therefore, we quantified PAA levels in the blood collected from the portal vein to assess their impact on the hepatic steatosis phenomes. The PAA levels were significantly lower in WD-fed *Shp*^*−/−*^ than in WT mice, consistent with their fatty liver phenotypes (Fig. 7A). Interestingly, PAA was reported to function as a PPARG agonist via direct binding and to activate its gene expression (53). Since low hepatic *Pparg2* mRNA levels are believed to contribute to the attenuation of the fatty liver phenotype in *Shp*^*−/−*^ mice (12), PAA involvement in *Pparg2* expression was assessed using a mouse hepatoma cell line (Fig. 7B). PAA treatment strongly induced the expression of *Pparg2* and its downstream target fat-specific protein 27 (*Fsp27*) within 5 h but not peroxisome proliferator-activated receptor gamma isoform 1 (*Pparg1*) or sterol regulatory element-binding protein 1 (*Srebp1*). To confirm these results in vivo, we assessed the mRNA levels of many other genes associated with PAA signaling in WT and *Shp*^*−/−*^ mice fed a WD. Figure 7C shows that the expression of positively associated genes (lipoprotein lipase [*Lpl*] and fatty acid binding protein 4 [*Fabp4*]) and *Pparg*2 and its target genes was significantly increased in WT mice upon WD challenge. However, their increases were significantly attenuated in *Shp*^*−/−*^ mice. In addition, negatively associated genes were downregulated only in WT but not in *Shp*^*−/−*^ mice fed a WD. Altogether, these results suggest that the regulatory role of orphan nuclear receptor SHP in BA synthesis affects metabolic phenomes, at least in part, by altering the gut microbiota, thereby decreasing fat absorption and serum levels of LPS and PAA, the two main gut bacterial products contributing to metabolic syndrome, mainly by enhancing gut barrier function and/or their production.

### Cohousing disrupts many metabolic parameters, including serum LPS and PAA levels observed in separately housed *Shp*^*−/−*^ mice

**Fig. 8:**
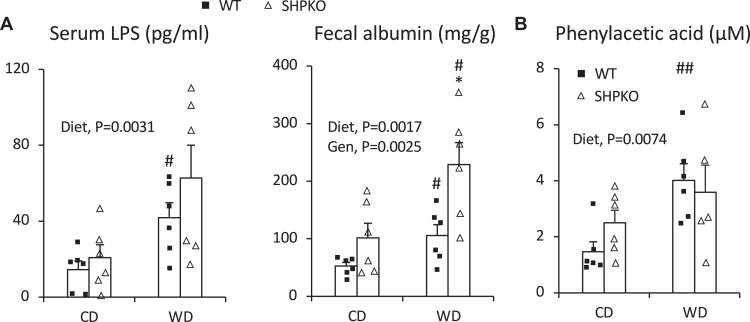
Serum LPS, fecal albumin, and serum PAA levels in cohoused littermate WT and *Shp*^*−/−*^ mice. A: Concentrations of serum LPS and fecal albumin were quantified in cohoused WT and *Shp*^*−/−*^ mice generated by heterozygous mating using commercial ELISA kits. The average values were plotted with SEM (*n* = 6). B: PAA levels were measured in the blood collected from portal veins of 4-month-old cohoused mice on 2 month diet regimen after 5 h fasting using a GC-MS. Average mean values were presented with SEM (*n* = 5–6). Two-way ANOVA was used to evaluate statistical significances. Student's *t*-test was also performed to evaluate statistical significance. #comparison with CD, ∗comparison with WT counterparts. Two symbols represent *P* < 0.01, and one symbol represents *P* < 0.05.

Serum PAA and LPS levels were also determined in cohoused mice to confirm their association with the phenotypes observed in the separately housed mice. Fig. 8A, B shows that LPS and PAA levels did not differ significantly between WT and *Shp*^*−/−*^ mice, especially those fed a WD. WD-fed *Shp*^*−/−*^ mice showed higher fecal albumin levels than the WT mice (Fig. 8A). The diet effect in two-way ANOVA was significantly higher in all WD-fed groups for all three parameters. The expression levels of intestinal and hepatic genes differentially regulated by *Shp* deletion were also assessed in cohoused mice. Intestinal genes involved in barrier function and TG absorption were similarly expressed in cohoused WT and *Shp*^*−/−*^ mice (Fig. 6D vs. C). Expression differences in hepatic genes associated with serum PAA between WT and *Shp*^*−/−*^ mice were partially or entirely blunted by cohousing (Fig. 7D vs. C), consistent with the disruption of partially protective phenotypes in cohoused *Shp*^*−/−*^ mice.

**Fig. 9:**
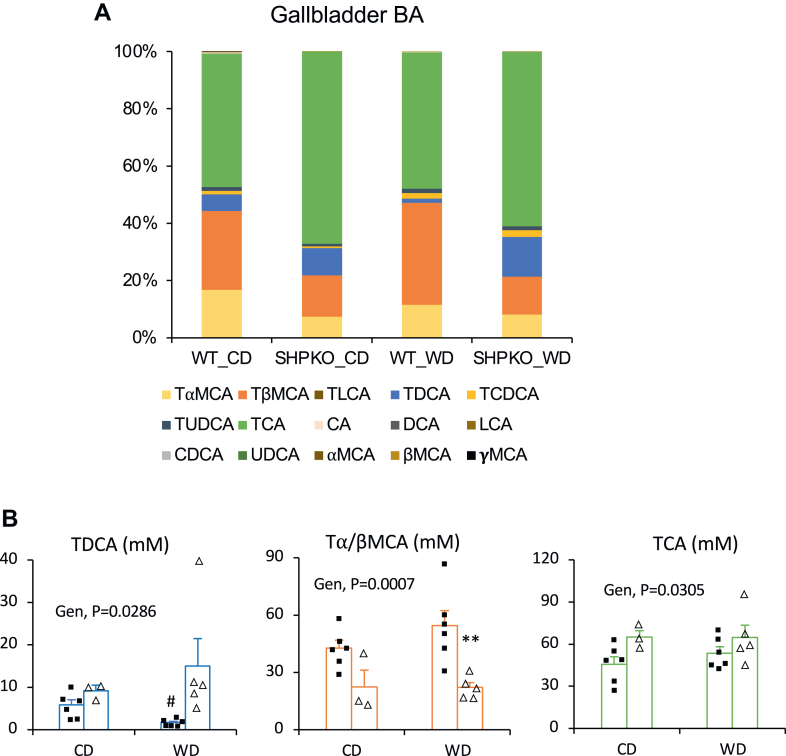
BA compositions in GBs of cohoused littermate animals. A: BA composition was determined in GBs of cohoused WT and *Shp*^*−/−*^ mice fed CD or WD. Their absolute concentrations were converted to percent values to be compared with values obtained from separately grouped animals in Fig. 2A. The average percent values of each BA species were presented as stacked bar graphs (*n* = WTCD [6], SHPKOCD [3], WTWD [6], and SHPKOWD [5]). B: Average concentrations of three major tauro-conjugated BAs were plotted with SEM values. Two-way ANOVA was performed to measure statistical significance. Student's *t*-test was also performed to compare two corresponding groups. #*P* < 0.05 compared with CD counterpart, ∗∗*P* < 0.01 compared with WT counterpart.

Since BA sharing between the cohoused WT and *Shp*^*−/−*^ mice would be expected to influence their BA composition, we analyzed the BA compositions in the GB bile from the cohoused WT and *Shp*^*−/−*^ mice (Fig. 9A). Each group’s BA composition was highly similar to that of corresponding separately housed mice (Fig. 2A), including the three major BAs, taurodeoxycholic acid, tauromuricholic acid, and TCA (Fig. 9B). We also analyzed the mRNA levels of major genes associated with BA synthesis and transport. supplemental Fig. S7 shows that the expression patterns of the major genes were almost indistinguishable between separately housed and cohoused mice. The coprophagic behavior of the mice appeared to have a minimal impact on BA composition change. Similar to hepatic TG, cohousing effectively disrupted the decrease in hepatic cholesterol level induced by *Shp* deletion in mice fed the WD, suggesting that amelioration of hepatic cholesterol in WD-fed *Shp*^*−/−*^ mice was also linked to changes in the gut microbiome but not changes in BA metabolism or profile. However, the mechanism linking the *Shp*^*−/−*^altered microbiome, LPS, PAA, and fecal albumin to changes in hepatic cholesterol metabolism remain to be determined (supplemental Fig. S8).

## Discussion

The orphan nuclear hormone receptor SHP has been identified as a transcriptional repressor that directly binds to numerous transcriptional activators, including other nuclear hormone receptors (4, 54). The repression is achieved by recruiting transcriptional repressors (55, 56). The suppression of *Cyp7a1* gene expression has been considered the major and well-studied SHP-driven transcriptional regulation. This critical regulation in BA metabolism has been confirmed in several mouse models, including *Shp*^*−/−*^ mice. Many mouse model studies have found that *Shp* deletion attenuates diet-induced obesity and NAFLD in the mice, whereas *Shp* overexpression has the opposite effect. While a few transcriptional targets responsible for these phenotypic manifestations have been proposed, the association of its BA metabolism with the observed metabolic phenotypes has not been studied or suggested. A recent study demonstrated that FXR (also called nuclear receptor subfamily 1, group H, member 4), a BA receptor, regulates obesity and fatty liver by altering the gut microbiota (20).

This study focused on assessing change in gut microbiota through the BA profile change and its impact on the partially protective phenotype against NASH development in *Shp*^*−/−*^ mice. Since DCA has been recognized as a major BA contributing to the inflammatory response and hepatocellular carcinoma (57), increased DCA levels in the serum and liver of *Shp*^*−/−*^ mice were unexpected and could not explain their phenotype. Therefore, altered gut microbiota composition because of *Shp* deletion was the major contributing factor to the phenotypic manifestations, which was proved by the cohousing scheme and gut microbiome structure determined by 16S rRNA gene sequencing analysis of fecal samples. The cohousing had little effect on BA composition in these mouse models. While phylum-level bacterial composition may agree with their metabolic phenotypes, many genus-level bacterial groups showed positive and negative correlations with phenotypes in previous studies. For example, one species in the genus *Bacteroides*, *Bacteroides vulgatus*, was overrepresented in mice resistant to diet-induced NAFLD (58). The genus *Bacteroides* was more abundant in *Shp*^*−/−*^ mice fed a WD than in WT mice, and its abundance was disrupted by cohousing. In contrast, while the genus *Dorea* was elevated in HFD-fed mice in an earlier study (59), this genus was not elevated by the WD in WT mice but was in *Shp*^*−/−*^ mice, resulting in a higher abundance in *Shp*^*−/−*^ mice than in WT mice fed a WD. In addition, the abundance of *Akkermansia muciniphila* was reported to inversely correlate with BW and diabetes, and treatment of mice with diet-induced obesity with this bacteria improved their adverse metabolic profile (60). Our study showed a higher abundance of the genus *Akkermansia* in *Shp*^*−/−*^ mice fed a WD than in WT mice housed in separate cages, consistent with the previous study. However, the difference in abundance was not reversed by cohousing, indicating that *Akkermansia muciniphila* may not contribute to the observed metabolic phenotypes in *Shp*^*−/−*^ mice, although our sequencing depth was limited to the genus level. Since similar discrepancies in bacterial association with metabolic disorders have been frequently documented in various studies (61), it is challenging to pinpoint specific bacteria responsible for the SHP-driven metabolic phenotypes with current approaches. Nevertheless, a few genera were found to differ significantly between WD-fed WT and *Shp*^*−/−*^ mice housed in separate cages but not when cohoused. Among these genera, Dehalobacterium in the phylum Firmicutes showed the strongest positive association with the phenotypic manifestations in the *Shp*^*−/−*^ mice, consistent with earlier studies (62, 63).

While BA composition and gut microbiota structure underlie the phenotype manifestations in *Shp*^*−/−*^ mice, the responsible pathways underlying their potential impacts remain unknown. The PICURSt assessment of 16S rRNA sequencing data showed that some enriched pathways in WT and *Shp*^*−/−*^ mice fed the WD correlated with the previously identified positive and negative pathways, respectively (16). These enriched pathways include flagellar assembly and bacterial motility proteins for WT mice and aromatic amino acid biosynthesis, β-alanine metabolism, pyruvate metabolism, and citrate cycle for *Shp*^*−/−*^ mice. The associations between these pathways and metabolic outcomes must be explored extensively to understand the underlying mechanisms and establish concrete connections.

Our study’s significant findings were the decreased serum LPS and PAA levels in *Shp*^*−/−*^ mice fed a WD compared with WT mice. In addition, hepatic *Pparg2* expression was shown to be a direct positive target of PAA. *Shp* deletion provides better circulatory environments to cope with WD-induced obesity and NASH by modifying the BA profile and gut microbiota. Chronic endotoxemia, represented by high bacterial LPS levels in the blood, is a well-known trigger for the onset of metabolic syndromes, including obesity, diabetes, and NASH (18). Notably, the Gram-negative phylum Proteobacteria was greatly increased in WT mice fed the WD compared with *Shp*^*−/−*^ mice. PAA is a recently identified phenylalanine metabolite in blood circulation strongly associated with steatosis among the microbial-mammalian cometabolites in patients with hepatic steatosis (25). In their studies, *LPL* and *FABP4*, two major targets of PPARγ2 (64), were among the strongly induced hepatic genes in patients with steatosis. While not normally expressed in the liver, these two genes were highly induced in the livers of human patients with NAFLD or mice fed a WD, along with *PPARG2* (65, 66, 67). The proteins encoded by these genes are essential components for the adipogenic program in adipose tissues. Given their specific roles in fat storage, surplus fats delivered to the liver by an HFD would be stored rather than oxidized or packaged into very low-density lipoprotein by activating these proteins. The direct link between bacterial metabolite PAA and hepatic PPARγ2 activation would be the first step for NASH development under the two-hit theory (53, 68).

Higher serum LPS induces inflammation in the steatotic liver caused concomitantly by PAA, eventually developing into NASH when persistently fed a WD. The bacterial gene responsible for PAA production from phenylalanine is mapped to several species belonging to the genus *Clostridium* (69). Unfortunately, the contribution of this genus to phenotypes in our experimental mice remains unknown because of variations in the abundance of various Clostridium species in our 16S rRNA gene sequencing analysis. An earlier study reported that PAA functions as a ligand for PPARγ and transcriptionally activates *Pparg* expression simultaneously (53). Therefore, we hypothesize that PAA-bound PPARγ1 (the abundant isoform in the liver) can activate the expression of another isoform (*Pparg2*). PPARγ binds direct repeat 1 elements in the nuclear receptor binding site with the retinoid X receptor. Our previous study identified a possible direct repeat 1 element in the *Pparg2* promoter that is bound by the hepatocyte nuclear factor 4 alpha-hairy and enhancer of split 6 to repress expression (70). We hypothesize that under normal conditions, the hepatocyte nuclear factor 4 alpha-hairy and enhancer of split 6 complex represses *Pparg2* expression, and increased PAA under an HFD may relieve this repression via activated PPARγ1 binding to induce an adipogenic program in the liver, inducing the expression of *Pparg2* and its downstream target genes. Hepatic TG synthesis rate was not measured in the present study to corroborate this hypothesis.

In summary, *Shp* deletion reshapes gut microbiome especially in WD-fed condition, in such a way as to (1) enhance gut barrier function and/or (2) reduce LPS and PAA production in the gut. This study also demonstrated that hepatic cholesterol is positively associated with NAFLD/NASH development. All these molecular responses to dietary-modified gut microbiota contribute to the development of obesity and NASH. While a germ-free mouse approach is necessary to provide a definitive conclusion, our cohousing data suggest that *Shp*^*−/−*^ mice show reduced molecular responses possibly because of BA-mediated dysbiosis, which remains to be determined.

## Data Availability

All data are contained within this article.

## Supplemental data

This article contains supplemental data (11, 16).

## Conflict of interest

The authors declare that they have no conflicts of interest with the contents of this article.
