# Supplementary material for: Microbial products linked to steatohepatitis are reduced by deletion of nuclear hormone receptor SHP in mice
Source: J Lipid Res. 2023 Nov 2;64(12):100469. doi: 10.1016/j.jlr.2023.100469 (PMC10698000; doi:10.1016/j.jlr.2023.100469)
Supplement: Supporting materials [file mmc1.pdf]

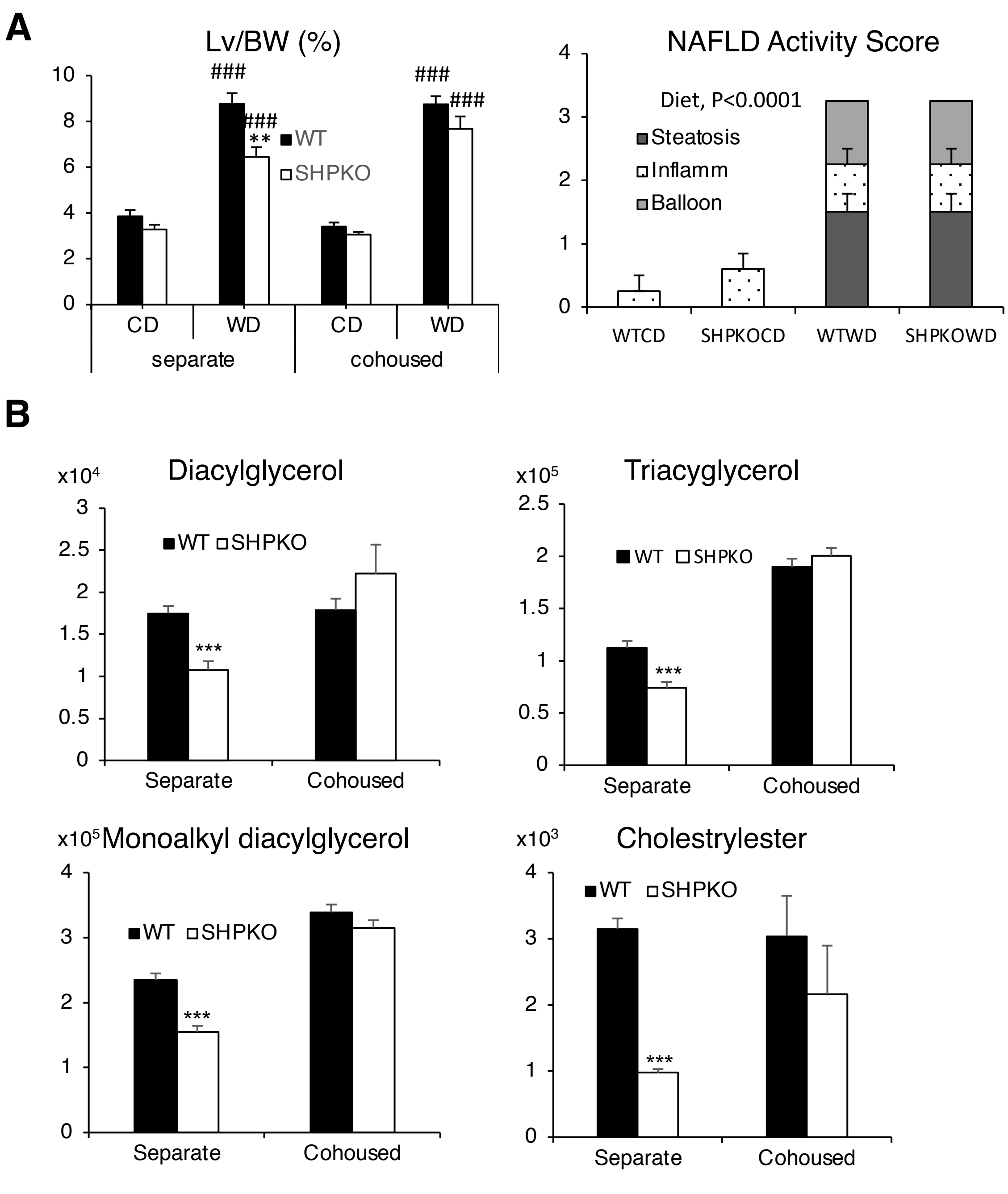

Supplemental Fig. S1. Cohousing disrupts the protection against hepatic steatosis in *Shp*$^{-/-}$ mice. (A) Experimental animals from main figure 1 were used to measure liver to body weight ratio after diet regimen (left). NAFLD activity scores of cohoused mouse live sections were evaluated and presented as means±SEM (right, n=4-5).Two-way ANOVA result was shown (B) Four hepatic lipid species significantly affected by cohousing in *Shp*$^{-/-}$ mice were replotted from the lipidomics obtained in main Fig. 1D.

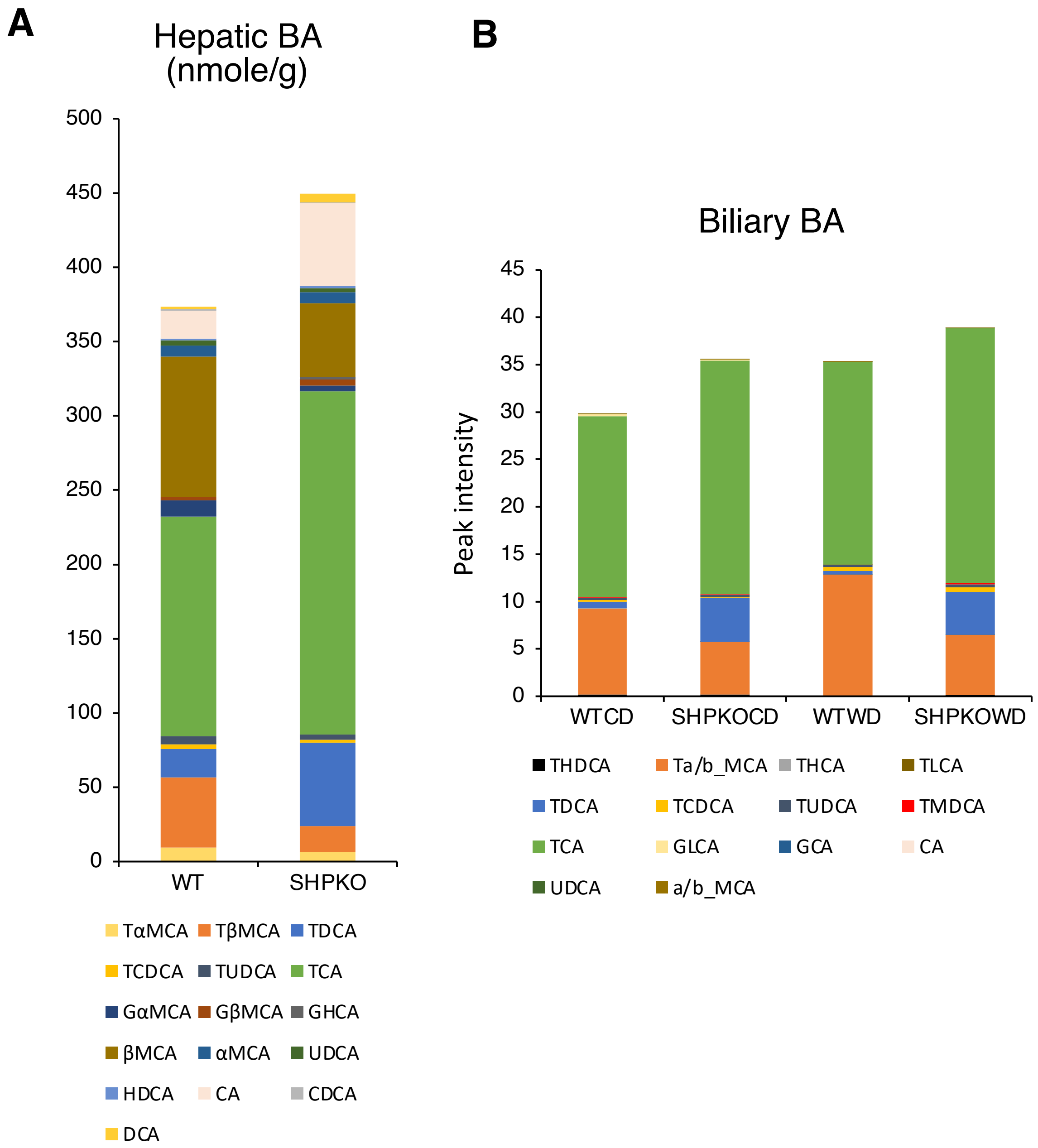

Supplemental Fig. S2. Hepatic and biliary bile acid composition in *Shp*$^{-/-}$ mice. (A) Detailed bile acid compositions including conjugated forms from livers of WT and *Shp*$^{-/-}$ mice fed chow were presented from data obtained in an earlier study (11). (B) Biliary BA composition was replotted based on absolute intensities from data in main Fig. 2A.

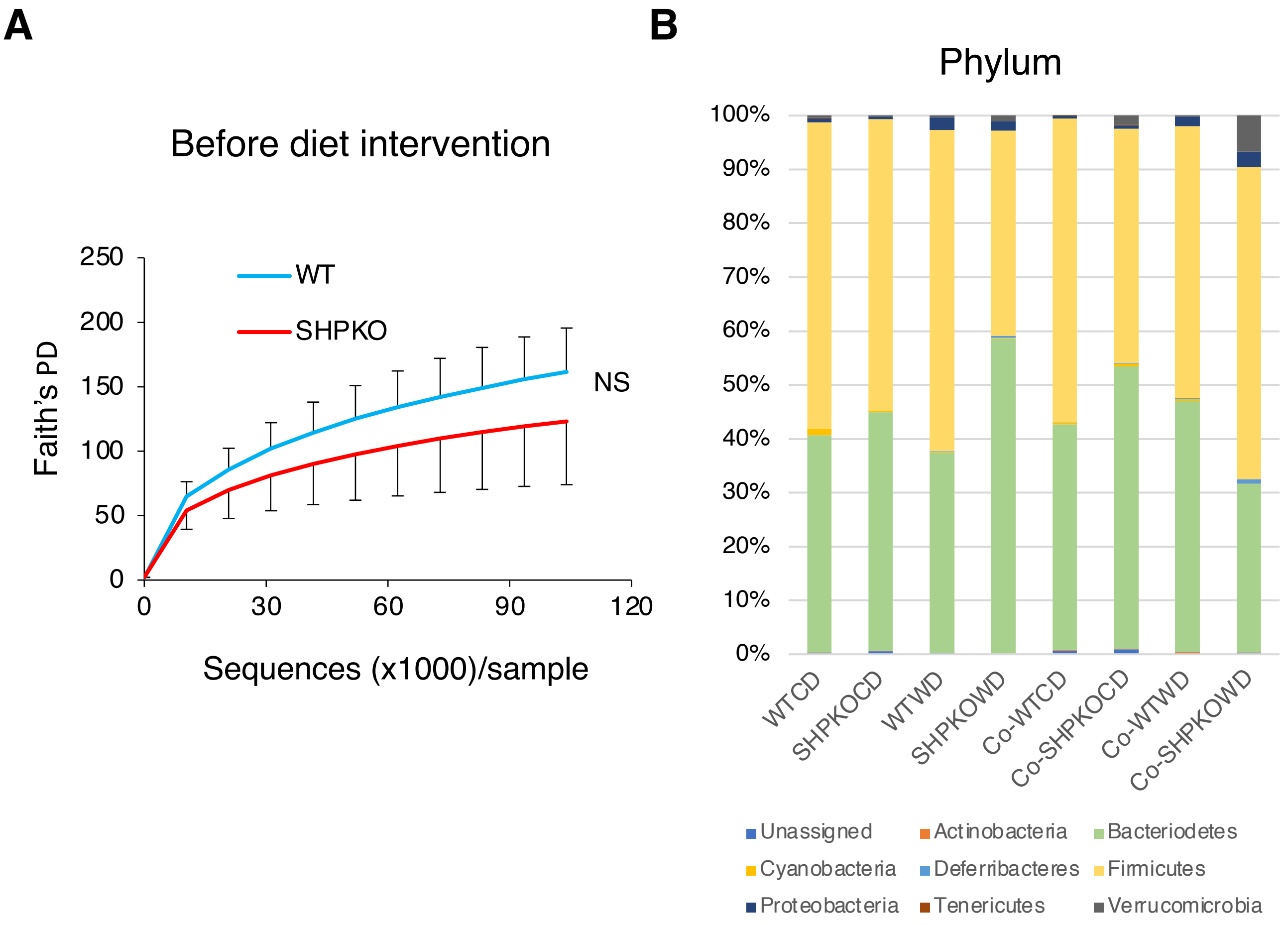

Supplemental Fig. S3. Fecal microbiome analysis in *Shp*$^{-/-}$ mice. (A) Faith's phylogenetic diversity was plotted based on fecal 16S rRNA gene sequencing data obtained 10 wk old WT and *Shp*$^{-/-}$ mice before WD regimen. (B) Phylum level gut microbes were plotted as percent values in each group of animals obtained from feces collected at 10 wk diet regimen.

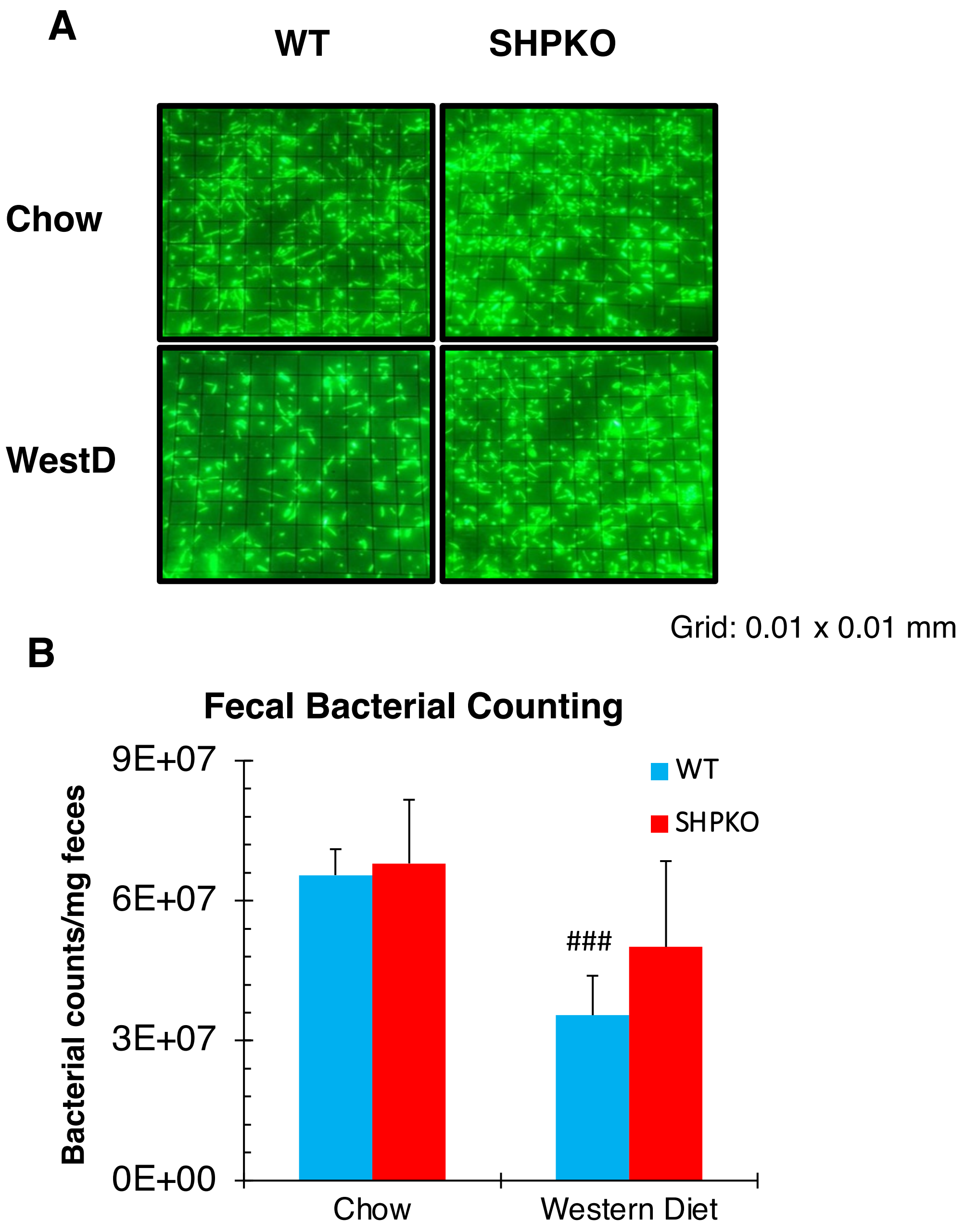

Supplemental Fig. S4. Fecal bacterial population in *Shp*$^{-/-}$ mice. (A) Fecal bacterial population in WT and *Shp*$^{-/-}$ mice was visualized using SYBR Green I fluorescent dye staining with 100X magnification. (B) Bacterial counting was performed using 10 microscopic images per animal and was plotted as mean counts per mg feces ± SEM (n=4).

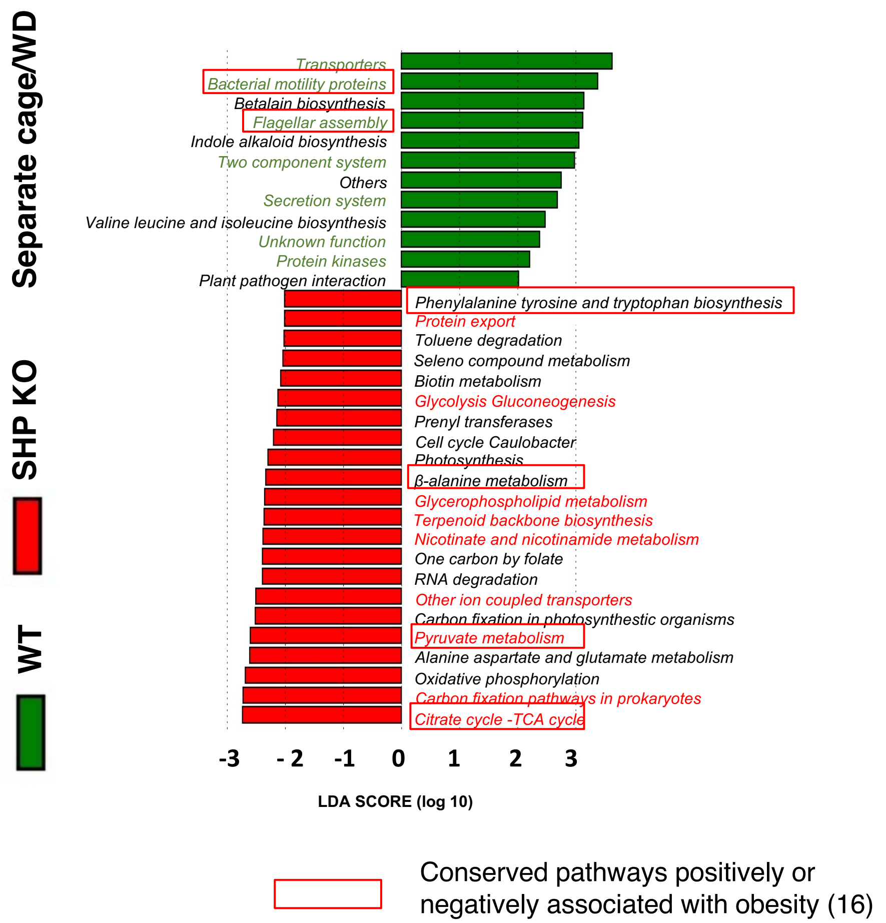

Supplemental Fig. S5. Enriched microbial functional compositions predicted from 16S rRNA gene sequencing data. Linear discriminant analysis effect size (LEfSe) was used to discriminate predicted functional biomarkers between WT and *Shp*$^{-/-}$ gut microbial OTUs on WD. Positively and negatively associated biomarkers with obesity are boxed with red rectangle based on an earlier report (16). Colored functions (green or red) are conserved with chow-fed counterparts, respectively.

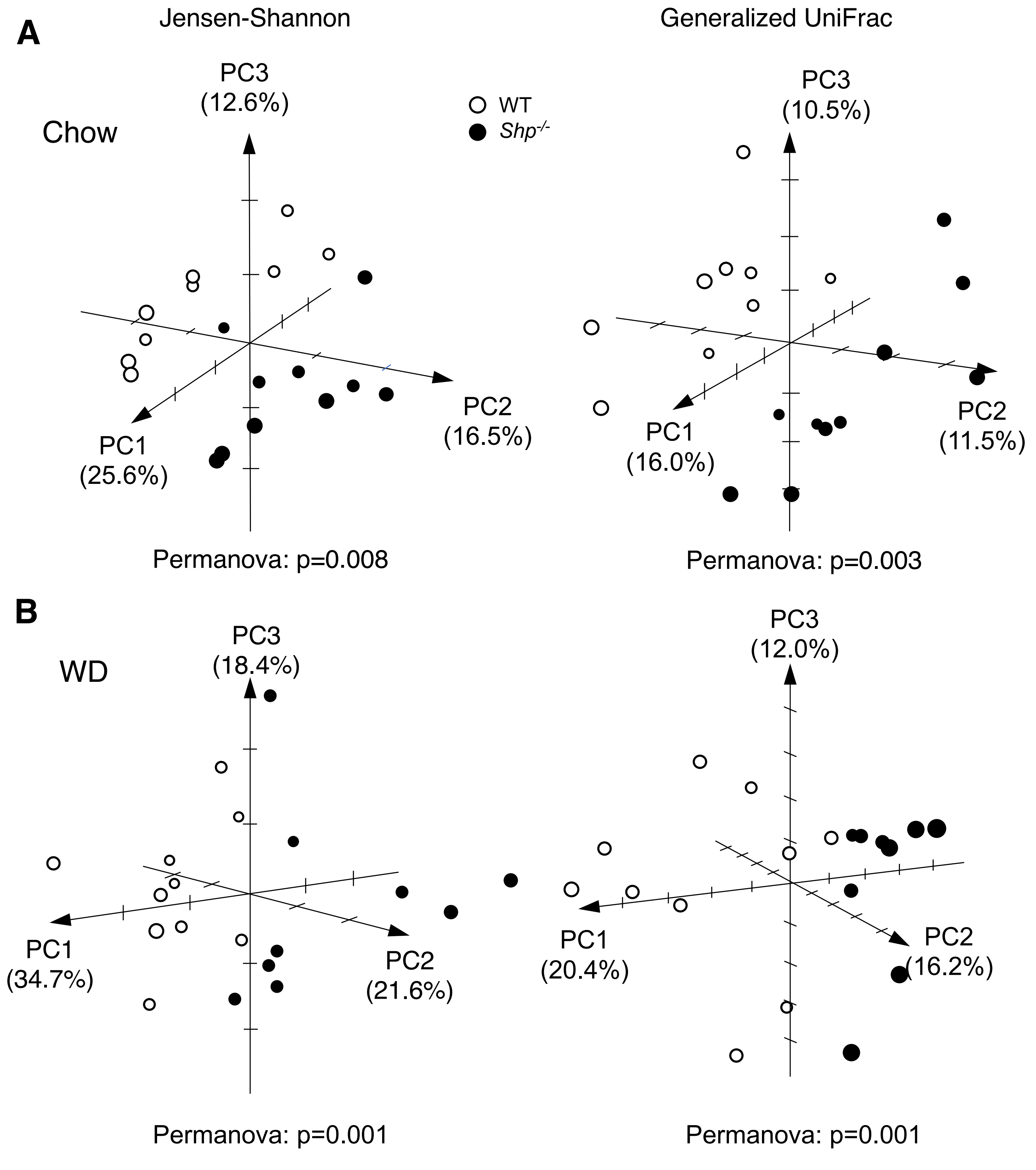

Supplemental Fig. S6. Gut dysbiosis is evident in *Shp*$^{-/-}$ littermates generated from heterozygous mating. PCoA plot was obtained by Jensen-Shanon (left panel) or Generalized UniFrac approach (right panel) using species level taxonomy identified in fecal DNA from WT and *Shp*$^{-/-}$ littermates fed chow (A) or WD (B).

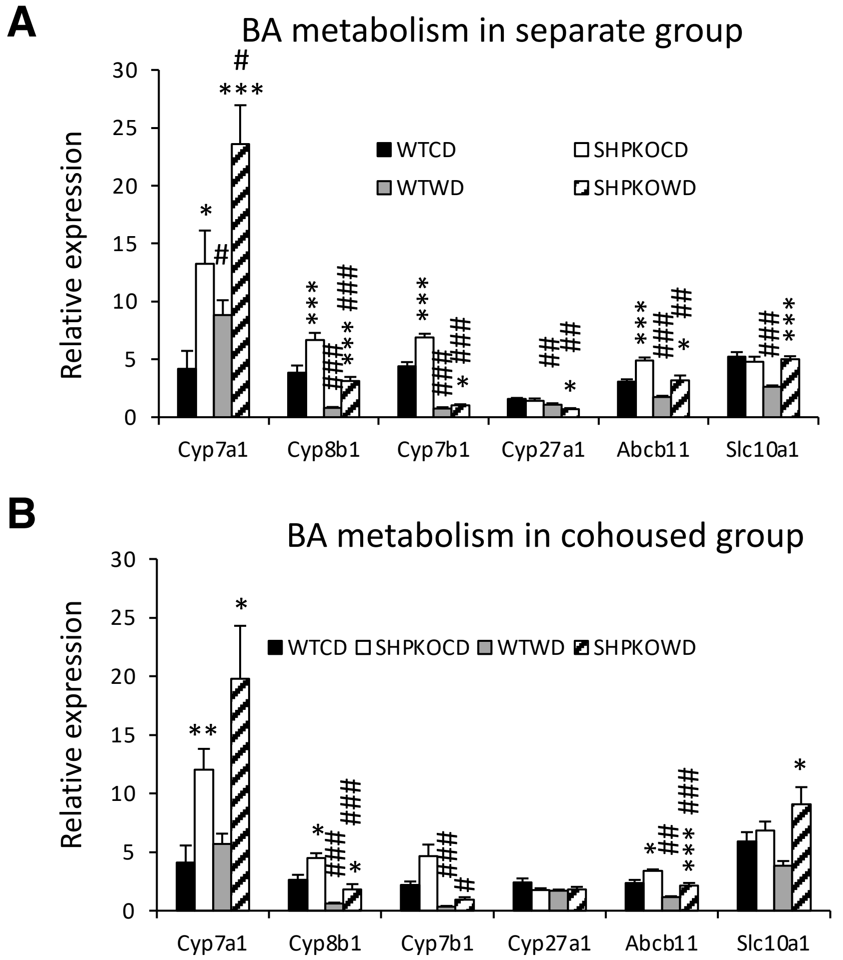

Supplemental Fig. S7. Expression of hepatic genes involved in bile acid synthesis and transportation. Realtime qPCR analysis was performed to quantify mRNA levels of the indicated genes in BA metabolism in the livers of WT and *Shp*$^{-/-}$ littermates fed chow or WD from (A) segregated group-housing and (B) cohousing strategies.

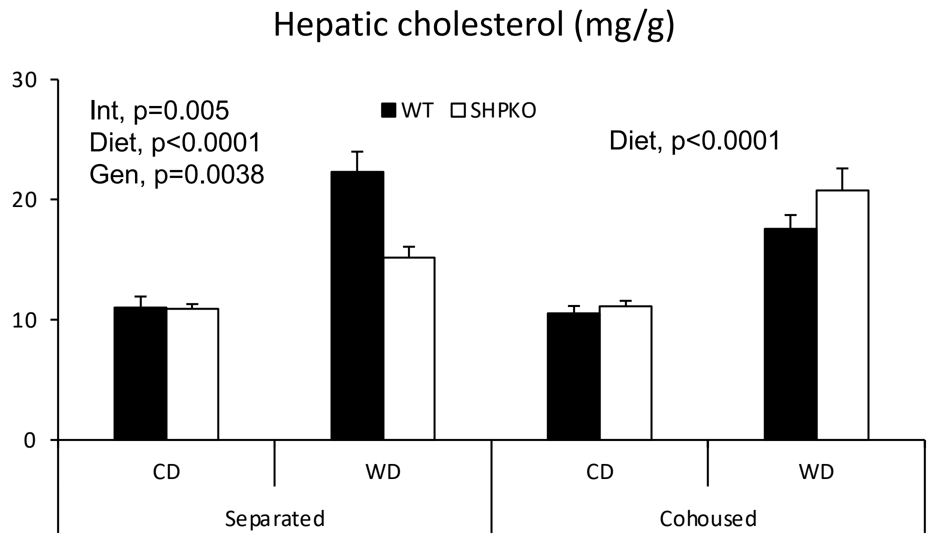

Supplemental Fig. S8. Cohousing disrupts the lowering cholesterol effect by SHP deletion. Cholesterol level was determined in the livers of WT and *Shp*$^{-/-}$ littermates fed chow (CD) or WD from segregated group-housing and cohousing strategies. The values were presented as mean ± SEM. Two-way ANOVA was performed for statistic analysis of each housing strategy. N=6-9 for separate group, n=6 for cohoused group

Supplemental Table 1. The bacterial 16S rRNA specific primers used for amplification (only sequences targeting rRNA are presented. M is for A or C; V is for A, C, or G; W is for A or T)

Forward primer	5'-GTGCCAGCMGCCGCGGTAA-3'
Reverse primer	5'-GGACTACHVGGGTWTCTAAT-3'
